# Neurological development in children born moderately or late preterm: national cohort study

**DOI:** 10.1136/bmj-2023-075630

**Published:** 2024-01-24

**Authors:** Ayoub Mitha, Ruoqing Chen, Neda Razaz, Stefan Johansson, Olof Stephansson, Maria Altman, Jenny Bolk

**Affiliations:** 1 Division of Clinical Epidemiology, Department of Medicine Solna, Karolinska Institutet, Stockholm, Sweden; 2 CHU Lille, Pediatric and Neonatal Intensive Care Transport Unit, Department of Emergency Medicine, Lille, France; 3 Université Paris Cité, CRESS, Obstetrical, Perinatal and Pediatric Epidemiology Research Team (EPOPé) INSERM, INRAE, Paris, France; 4 School of Public Health (Shenzhen), Sun Yat-sen University, Shenzhen, 518107, China; 5 Institute of Environmental Medicine, Karolinska Institutet, Stockholm, Sweden; 6 Department of Clinical Science and Education, Södersjukhuset, Karolinska Institutet, Stockholm, Sweden; 7 Sachs’ Children and Youth Hospital, Södersjukhuset, Stockholm, Sweden; 8 Department of Pediatric Rheumatology, Astrid Lindgren Children’s Hospital, Karolinska University Hospital, Stockholm, Sweden

## Abstract

**Objective:**

To assess long term neurodevelopmental outcomes of children born at different gestational ages, particularly 32-33 weeks (moderately preterm) and 34-36 weeks (late preterm), compared with 39-40 weeks (full term).

**Design:**

Nationwide cohort study.

**Setting:**

Sweden.

**Participants:**

1 281 690 liveborn singleton children without congenital malformations born at 32^+0^ to 41^+6^ weeks between 1998 and 2012.

**Main outcome measures:**

The primary outcomes of interest were motor, cognitive, epileptic, hearing, and visual impairments and a composite of any neurodevelopmental impairment, diagnosed up to age 16 years. Hazard ratios and 95% confidence intervals were estimated using Cox regression adjusted for parental and infant characteristics in the study population and in the subset of full siblings. Risk differences were also estimated to assess the absolute risk of neurodevelopmental impairment.

**Results:**

During a median follow-up of 13.1 years (interquartile range 9.5-15.9 years), 75 311 (47.8 per 10 000 person years) liveborn singleton infants without congenital malformations had at least one diagnosis of any neurodevelopmental impairment: 5899 (3.6 per 10 000 person years) had motor impairment, 27 371 (17.0 per 10 000 person years) cognitive impairment, 11 870 (7.3 per 10 000 person years) epileptic impairment, 19 700 (12.2 per 10 000 person years) visual impairment, and 20 393 (12.6 per 10 000 person years) hearing impairment. Children born moderately or late preterm, compared with those born full term, showed higher risks for any impairment (hazard ratio 1.73 (95% confidence interval 1.60 to 1.87) and 1.30 (1.26 to 1.35); risk difference 4.75% (95% confidence interval 3.88% to 5.60%) and 2.03% (1.75% to 2.35%), respectively) as well as motor, cognitive, epileptic, visual, and hearing impairments. Risks for neurodevelopmental impairments appeared highest from 32 weeks (the earliest gestational age), gradually declined until 41 weeks, and were also higher at 37-38 weeks (early term) compared with 39-40 weeks. In the sibling comparison analysis (n=349 108), most associations remained stable except for gestational age and epileptic and hearing impairments, where no association was observed; for children born early term the risk was only higher for cognitive impairment compared with those born full term.

**Conclusions:**

The findings of this study suggest that children born moderately or late preterm have higher risks of adverse neurodevelopmental outcomes. The risks should not be underestimated as these children comprise the largest proportion of children born preterm. The findings may help professionals and families achieve a better risk assessment and follow-up.

**Figure:**
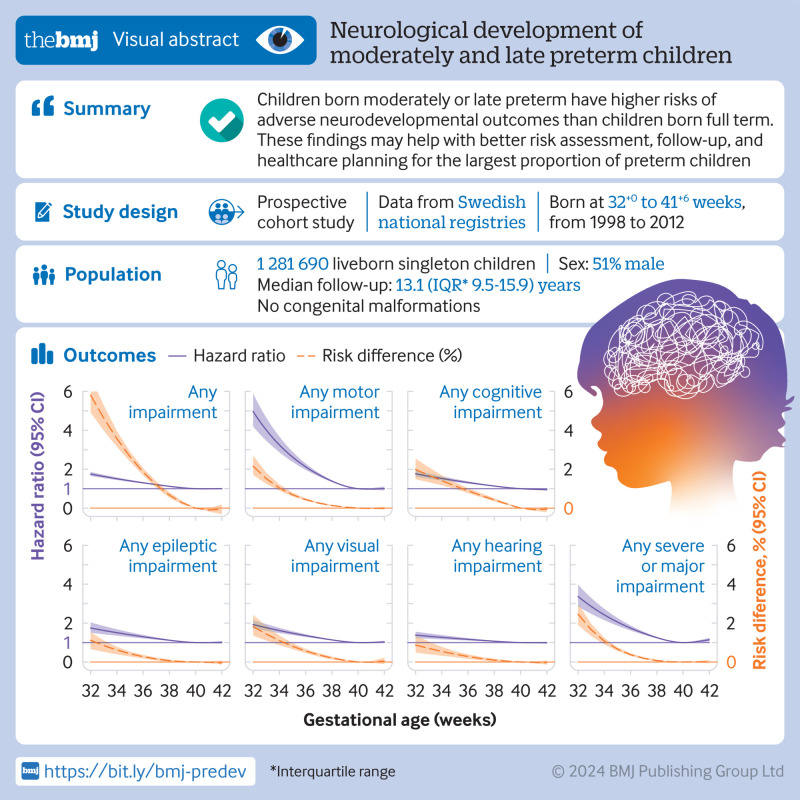


## Introduction

Children born preterm have higher risks of neurodevelopmental and behavioural disabilities in the first years of life and throughout childhood and adolescence compared with children born at term.^1^ Studies have mainly focused on the long term outcomes of children born extremely preterm (<28 weeks) or very preterm (28 to <32 weeks), despite the fact that children born moderately (32-33 weeks) or late (34-36 weeks) preterm account for about 80% of all children born preterm.^2^ ^3^ ^4^ ^5^

Children born moderately or late preterm represent a major healthcare burden in neonatal medicine,^6^ ^7^ and even small increases in adverse outcomes may have important consequences from a public health perspective, including the day-to-day functioning of children and their families. Recent reports indicate that compared with their peers born at term (≥37 weeks), children born moderately or late preterm are at higher risk of neurodevelopmental disabilities, with impaired cognition,^8^ ^9^ ^10^ ^11^ ^12^ ^13^ ^14^ ^15^ ^16^ impaired language^8^ ^10^ ^11^ ^15^ ^17^ ^18^ and motor function,^8^ ^10^ ^11^ ^15^ ^16^ ^19^ lower social-emotional competence,^8^ ^12^ ^13^ ^15^ ^20^ and higher risk of poor school performance.^13^ ^15^ ^21^ ^22^ ^23^ ^24^ ^25^ In contrast with studies of children born extremely preterm,^26^ ^27^ ^28^ ^29^ ^30^ ^31^ most studies of children born moderately or late preterm are not population based.^8^ ^9^ ^10^ ^11^ ^12^ ^13^ ^15^ ^17^ ^20^ ^21^ ^22^ ^23^ ^24^ ^25^ Population based studies are needed for more accurate risk estimates for children born moderately or late preterm, using standardised outcome measures and thus allowing follow-up of neurodevelopmental outcomes over time.^4^

In this nationwide cohort of more than one million liveborn singleton children of gestational age 32^+0^ weeks to 41^+6^ weeks, we assessed long term neurodevelopmental outcomes of children born at different gestational ages, particularly those born moderately or late preterm, compared with children born full term.

## Methods

### Data sources

Using the unique personal identity numbers of mothers and children,^32^ we linked data from the Swedish Medical Birth Register^33^ to several Swedish national registries: the National Patient Register,^34^ Total Population Register,^35^ Education Register,^36^ and Cause of Death Register.^37^ Extensive validation of the Medical Birth Register has shown high validity for most variables and coverage of prospectively collected information on almost all births in Sweden since 1973.^33^ The Swedish National Patient Register provides information on primary and secondary diagnoses at discharge for all patients admitted to hospital care since 1987 and from specialised outpatient care units since 2001.

### Study population

This population based cohort study included 1 496 950 births recorded in the Swedish Medical Birth Register from 1 January 1998 to 31 December 2012. We excluded stillbirths (n=5255), multiple births (n=43 602), children with major congenital malformations (n=51 858), births with missing information on personal identity number of children or mothers (n=1843), children with missing data on infant’s sex (n=7), children who emigrated (n=113) or died (n=2025) before age 28 days, children with missing data on gestational age (n=871), and children with gestational age <32 weeks (n=7616) and ≥42 weeks (n=102 070). After exclusions, the study population comprised 1 281 690 liveborn singleton children without congenital malformations born from 32^+0^ to 41^+6^ weeks (see supplementary figure A). Supplementary table A provides information on the ICD-10 (international classification of diseases and related health problems, 10th revision) codes for major congenital malformations.

### Gestational age

Gestational age (recorded in days) was determined using a hierarchy: early second trimester ultrasonography (88.4%), date of last menstrual period (6.6%), or postnatal assessment (4.9%).^33^ To analyse gestational age in weeks as a continuous variable, we divided the days by seven and rounded up to one decimal place. To analyse gestational age as a categorical variable, we rounded gestational age down to completed week and categorised children as born moderately preterm (32-33 weeks), late preterm (34-36 weeks), early term (37-38 weeks), full term (39-40 weeks), and late term (41 weeks).^7^

### Outcomes

We obtained information on neurodevelopmental outcomes, including motor, cognitive, epileptic, visual, and hearing impairments, from the Swedish National Patient Register. Each outcome was defined as at least one diagnosis of any of the outcomes in the register. A composite outcome of any neurodevelopmental impairment was defined as a diagnosis of one or more of motor, cognitive, epileptic, visual, or hearing impairment. A severe or major impairment was defined as a diagnosis of one or more of cerebral palsy, severe mental retardation, generalised epilepsy, and severe hearing or visual impairment. Supplementary table A provides information on ICD-10 codes for these outcomes. All children born from 1998 to 2012 were followed for each outcome from 28 days after birth until the date of first diagnosis of the neurodevelopmental outcome, death, emigration, 16th birthday, or 31 December 2019, whichever came first. Therefore, each child had a minimum of follow-up of seven years. Autism spectrum disorders and attention deficit/hyperactivity disorder were not included as outcomes in the current study because those outcomes based on data from Swedish registries have been published for preterm birth.^38^ ^39^ ^40^

### Covariates

Characteristics reported to be associated with both gestational age and neurodevelopmental impairments were considered as potential confounders based on a directed acyclic graph (see supplementary figure B). Maternal characteristics included age at delivery,^41^ ^42^ parity,^41^ ^43^ ^44^ country of birth,^41^ ^44^ cohabiting status,^41^ ^45^ body mass index (BMI) during early pregnancy,^46^ ^47^ and smoking during pregnancy.^37^ ^43^ ^48^ Maternal diseases included diabetic and hypertensive diseases.^2^ ^42^ ^44^ Parents’ characteristics included parental highest educational level and parental history of neurological or psychiatric disorder.^41^ ^44^ We also included information on calendar year of delivery to control for temporal changes in obstetric and neonatal practice and in diagnosis of neurodevelopmental outcomes.^49^ Characteristics of the infants included infant’s sex^44^ ^45^ and birth weight for gestational age, the latter being calculated based on the Swedish national sex specific reference curve for fetal growth.^41^ ^44^ ^50^ Supplementary table A provides the ICD-10 codes for parental diseases.

### Statistical analysis

Parental and infant characteristics were described among children born moderately preterm (32-33 weeks), late preterm (34-36 weeks), early term (37-38 weeks), full term (39-40 weeks), and late term (41 weeks). We calculated the incidence rates of each outcome studied during follow-up by gestational age group. The number of impaired neurodevelopmental outcomes among the affected children was also described.

To assess the association between gestational age and each outcome of interest, we used Cox proportional hazards regression to estimate hazard ratios along with 95% confidence intervals across the five gestational age groups, with 39-40 weeks as the reference, and between each completed week using 40 weeks as the reference. Age of the child was used as the underlying time scale. Schoenfeld residuals were used to test the proportional hazards assumption. We also estimated risk differences as P(X)−P(40), where P(X) is the risk of developing a neurodevelopmental outcome by age 16 years at a certain gestational age X, and P(40) is the corresponding risk at 40 weeks of gestation (reference). To consider the impact of preterm birth on the neurodevelopmental health of the population, we further estimated the population attributable fraction, defined as the proportion of the cases of neurodevelopmental impairment in the entire population attributable to a specific gestational age group, instead of 39-40 gestational weeks. Hazard ratios, risk differences, and population attributable fractions along with the corresponding 95% confidence intervals were adjusted for maternal characteristics (age at delivery, parity, country of birth, cohabiting status, BMI during early pregnancy, smoking during pregnancy, calendar period of delivery), maternal diseases (diabetic and hypertensive diseases), parental characteristics (highest educational level and history of neurological or psychiatric disorder), and birth characteristics of the infants (sex and birth weight for gestational age). In addition, to assess the potential non-linear relationship of each outcome with gestational age on a continuous scale, we used restricted cubic splines with three knots positioned at the 10th, 50th, and 90th centiles of the distribution of the gestational age variable; the hazard ratios and risk differences were estimated using 40^+0^ completed gestational weeks as the reference. To assess the impact of birth weight for gestational age on long term outcomes among children born moderately or late preterm, we estimated hazard ratios stratified by birth weight for gestational age categories among children born preterm. Finally, to account for the correlation among full siblings, we used a robust sandwich estimator to correct standard errors in the analyses.

We performed several sensitivity analyses, estimating hazard ratios for the studied associations. Firstly, because we used complete case analysis in the primary analysis, results might have been biased owing to missing values of confounders (missing proportions in the variables ranging from <0.1% to 10.9%). We therefore conducted the Cox regression analysis using multiple imputation of missing values with chained equations.^51^ Ten imputations with 50 iterations each were implemented, and the imputation was informed using maternal characteristics, maternal diseases, parental characteristics, birth characteristics of infants, gestational age, and each outcome of interest. Secondly, we performed a sibling comparison analysis to control for unmeasured shared genetic and environmental factors. In this analysis, only full siblings discordant for both gestational age (ie, siblings in different gestational age groups) and outcome (ie, siblings with different time to event) were informative and thus were included. Stratified Cox regression was conducted and adjusted for confounding factors except maternal country of birth and parental educational level. Thirdly, we investigated if the level of risk differed by type of onset of labour (spontaneous versus induced) using formal tests for interaction. Fourthly, because of the difference in coverage of calendar years between inpatient and outpatient data in the National Patient Register, we performed an analysis in which we restricted the population to children born from 2001 to 2012, when data on both hospital admission and outpatient care were available.

Data management and preparation were performed using SAS version 9.4 (SAS Institute, Cary, NC). Statistical analyses were performed using Stata version 15.1 (StataCorp, College Station, TX) and R version 4.1.3 (R Foundation for Statistical Computing, Vienna, Austria).

### Patient and public involvement

Although we support the importance of patient and public involvement, this study was based on analysis of information available from linkage of anonymised data in national registries. No patients were directly involved in designing the research question or the outcome measures, nor were they involved in developing plans for implementation of the study. No patients were asked to advise on interpretation or writing up of results. The collection of patient data in national healthcare registries in Sweden dates back to the 1970s, when patient and public engagement in healthcare and research was less common. As yet, there are no structured processes in Sweden around those data sources, and how national authorities, professional organisations, and research departments are to manage patient and public involvement. This study also lacked funding for patient and public involvement. However, the impetus for this study was parental concerns about follow-up care of moderately and late preterm infants often expressed by families during their stay in the neonatal intensive care unit.

## Results

Of 1 281 690 liveborn singleton children, 7525 (0.6%) were born at 32-33 weeks, 48 772 (3.8%) at 34-36 weeks, 257 591 (20.1%) at 37-38 weeks, 713 952 (55.7%) at 39-40 weeks, and 253 850 (19.8%) at 41 weeks. Parental characteristics that were more common in children born moderately or late preterm compared with children born full term were young maternal age (<25 years) at delivery, primiparity, mother not cohabiting with partner, maternal obesity (BMI ≥35), maternal smoking during pregnancy, maternal diabetic and hypertensive diseases, parental low (<12 years) educational level, and parental history of neurological or psychiatric disorder (table 1). Children born preterm more often had a low birth weight for gestational age (<10th centile), and male sex was overrepresented (table 1).

**Table 1:** Characteristics of parents and of liveborn singleton children of gestational age 32-41 weeks without congenital malformations in Sweden 1998-2012. Values are number (column percentage) unless stated otherwise

Characteristics	Total	Gestational age (weeks)				
		32-33	34-36	37-38	39-40	41
Total*	1 281 690 (100.0)	7525 (0.6)	48 772 (3.8)	257 591 (20.1)	713 952 (55.7)	253 850 (19.8)
**Mothers**						
Age at delivery (years):						
<20	21 611 (1.7)	184 (2.4)	1019 (2.1)	4461 (1.7)	12 175 (1.7)	3772 (1.5)
20-24	168 322 (13.1)	1034 (13.7)	7132 (14.6)	32 516 (12.6)	95 661 (13.4)	31 979 (12.6)
25-29	393 511 (30.7)	2271 (30.2)	15 096 (31.0)	75 696 (29.4)	223 145 (31.3)	77 303 (30.5)
30-34	444 025 (34.6)	2394 (31.8)	15 548 (31.9)	87 745 (34.1)	248 012 (34.7)	90 326 (35.6)
≥35	254 221 (19.8)	1642 (21.8)	9977 (20.5)	57 173 (22.2)	134 959 (18.9)	50 470 (19.9)
Parity:						
1	555 625 (43.4)	4272 (56.8)	26 171 (53.7)	105 201 (40.8)	301 246 (42.2)	118 735 (46.8)
2-3	653 680 (51.0)	2759 (36.7)	19 455 (39.9)	134 937 (52.4)	374 278 (52.4)	122 251 (48.2)
≥4	72 385 (5.6)	494 (6.6)	3146 (6.5)	17 453 (6.8)	38 428 (5.4)	12 864 (5.1)
Country of birth:						
Nordic†	1 043 737 (81.4)	6124 (81.4)	39 934 (81.9)	205 647 (79.8)	580 261 (81.3)	211 771 (83.4)
Other	237 540 (18.5)	1395 (18.5)	8822 (18.1)	51 858 (20.1)	133 466 (18.7)	41 999 (16.5)
Missing	413 (0.0)	6 (0.1)	16 (0.0)	86 (0.0)	225 (0.0)	80 (0.0)
Cohabiting:						
Yes	1 149 088 (89.7)	6296 (83.7)	42 224 (86.6)	229 006 (88.9)	642 584 (90.0)	228 978 (90.2)
No	68 015 (5.3)	548 (7.3)	3051 (6.3)	14 396 (5.6)	36 667 (5.1)	13 353 (5.3)
Missing	64 587 (5.0)	681 (9.0)	3497 (7.2)	14 189 (5.5)	34 701 (4.9)	11 519 (4.5)
Early pregnancy BMI:						
<18.5	27 658 (2.2)	204 (2.7)	1327 (2.7)	6547 (2.5)	15 490 (2.2)	4090 (1.6)
18.5-24.9	702 823 (54.8)	3686 (49.0)	24 639 (50.5)	137 606 (53.4)	399 828 (56.0)	137 064 (54.0)
25-29.9	285 315 (22.3)	1640 (21.8)	10 666 (21.9)	56 434 (21.9)	156 949 (22.0)	59 626 (23.5)
30-34.9	90 295 (7.0)	586 (7.8)	3835 (7.9)	19 039 (7.4)	47 757 (6.7)	19 078 (7.5)
35-39.9	26 746 (2.1)	191 (2.5)	1321 (2.7)	6168 (2.4)	13 492 (1.9)	5574 (2.2)
≥40	9347 (0.7)	81 (1.1)	514 (1.1)	2149 (0.8)	4649 (0.7)	1954 (0.8)
Missing	139 506 (10.9)	1137 (15.1)	6470 (13.3)	29 648 (11.5)	75 787 (10.6)	26 464 (10.4)
Smoking during pregnancy:						
No	1 107 880 (86.4)	5917 (78.6)	39 946 (81.9)	218 594 (84.9)	620 483 (86.9)	222 940 (87.8)
Yes	113 881 (8.9)	908 (12.1)	5409 (11.1)	25 641 (10.0)	61 581 (8.6)	20 342 (8.0)
Missing	59 929 (4.7)	700 (9.3)	3417 (7.0)	13 356 (5.2)	31 888 (4.5)	10 568 (4.2)
Diabetic diseases:						
No	1 262 470 (98.5)	7259 (96.5)	46 876 (96.1)	250 940 (97.4)	705 148 (98.8)	252 247 (99.4)
Pregestational diabetes	5909 (0.5)	142 (1.9)	924 (1.9)	2552 (1.0)	2108 (0.3)	183 (0.1)
Gestational diabetes	13 311 (1.0)	124 (1.6)	972 (2.0)	4099 (1.6)	6696 (0.9)	1420 (0.6)
Hypertensive diseases:						
No	1 238 394 (96.6)	5975 (79.4)	43 131 (88.4)	244 889 (95.1)	695 988 (97.5)	248 411 (97.9)
Pregestational hypertension	8297 (0.6)	178 (2.4)	713 (1.5)	2282 (0.9)	3965 (0.6)	1159 (0.5)
Pre-eclampsia	34 999 (2.7)	1372 (18.2)	4928 (10.1)	10 420 (4.0)	13 999 (2.0)	4280 (1.7)
Calendar period of delivery:						
1998-2002	379 175 (29.6)	2306 (30.6)	14 968 (30.7)	75 056 (29.1)	210 271 (29.5)	76 574 (30.2)
2003-07	430 912 (33.6)	2615 (34.8)	16 490 (33.8)	89 330 (34.7)	237 644 (33.3)	84 833 (33.4)
2008-12	471 603 (36.8)	2604 (34.6)	17 314 (35.5)	93 205 (36.2)	266 037 (37.3)	92 443 (36.4)
**Parents**						
Highest educational level (years):						
≤11	174 172 (13.6)	1249 (16.6)	7700 (15.8)	38 668 (15.0)	94 672 (13.3)	31 883 (12.6)
12-14	525 372 (41.0)	3227 (42.9)	20 885 (42.8)	107 285 (41.6)	291 425 (40.8)	102 550 (40.4)
≥15	580 487 (45.3)	3042 (40.4)	20 145 (41.3)	111 272 (43.2)	326 957 (45.8)	119 071 (46.9)
Missing	1659 (0.1)	7 (0.1)	42 (0.1)	366 (0.1)	898 (0.1)	346 (0.1)
History of neurological or psychiatric disorder:						
No	1 120 660 (87.4)	6390 (84.9)	41 344 (84.8)	220 040 (85.4)	627 805 (87.9)	225 081 (88.7)
Yes	161 030 (12.6)	1135 (15.1)	7428 (15.2)	37 551 (14.6)	86 147 (12.1)	28 769 (11.3)
**Infants**						
Sex:						
Female	633 069 (49.4)	3321 (44.1)	22 790 (46.7)	128 438 (49.9)	359 204 (50.3)	119 316 (47.0)
Male	648 621 (50.6)	4204 (55.9)	25 982 (53.3)	129 153 (50.1)	354 748 (49.7)	134 534 (53.0)
Birth weight for gestational age (centiles):						
<3rd	27 650 (2.2)	1094 (14.5)	2933 (6.0)	6346 (2.5)	12 422 (1.7)	4855 (1.9)
3rd-10th	77 471 (6.0)	862 (11.5)	3484 (7.1)	14 270 (5.5)	41 663 (5.8)	17 192 (6.8)
10th-90th	1 045 759 (81.6)	4849 (64.4)	36 139 (74.1)	203 105 (78.8)	590 965 (82.8)	210 701 (83.0)
90th-97th	80 494 (6.3)	244 (3.2)	2975 (6.1)	19 084 (7.4)	44 077 (6.2)	14 114 (5.6)
≥97th	46 953 (3.7)	280 (3.7)	2886 (5.9)	14 038 (5.4)	23 342 (3.3)	6407 (2.5)
Missing	3363 (0.3)	196 (2.6)	355 (0.7)	748 (0.3)	1483 (0.2)	581 (0.2)

BMI=body mass index.

* Numbers and row percentages.

† Includes Sweden, Denmark, Finland, Iceland, and Norway.

The total and median follow-up time was 15 772 478.4 person years and 13.1 (interquartile range 9.5-15.9) years, respectively. Overall, 75 311 (47.8 per 10 000 person years) children had any neurodevelopmental impairment, most first diagnosed in specialised outpatient care (see supplementary table B). Of those, 5899 (3.6 per 10 000 person years) had motor impairment, 27 371 (17.0 per 10 000 person years) cognitive impairment, 11 870 (7.3 per 10 000 person years) epileptic impairment, 19 700 (12.2 per 10 000 person years) visual impairment, and 20 393 (12.6 per 10 000 person years) hearing impairment. Severe or major impairment was diagnosed in 8052 children (5.0 per 10 000 person years). A total of 1890 (0.1%) children died during follow-up. Children with diagnoses of neurodevelopmental outcomes mainly presented with one impairment (see supplementary table C).

**Fig 1:**
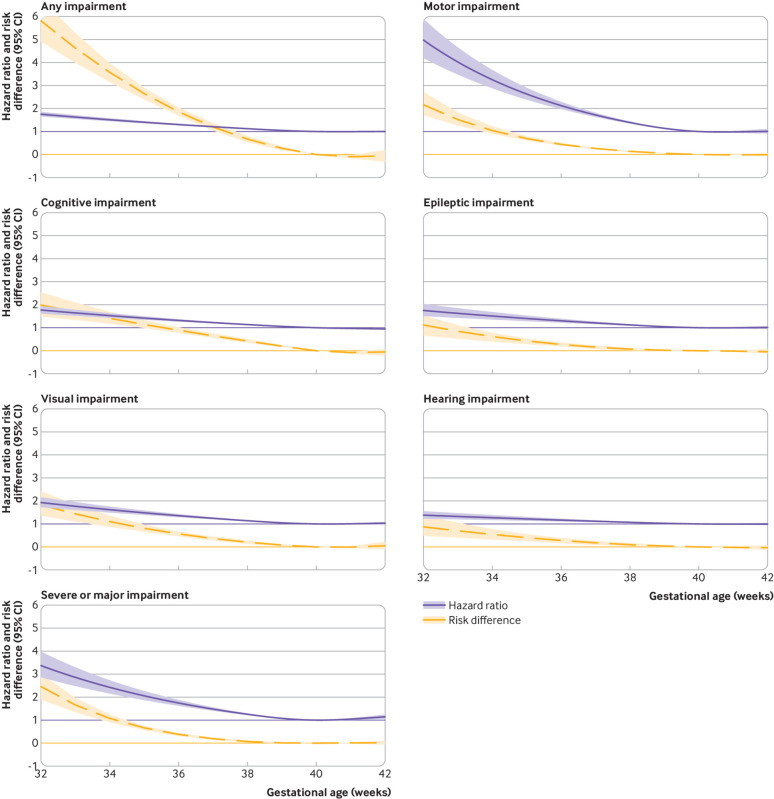
Association between gestational age and neurodevelopmental outcomes among liveborn singleton children without congenital malformations in Sweden 1998-2012. Risk difference is the difference in risk of neurodevelopmental outcome by age 16 years comparing different gestational ages. Hazard ratios and risk differences are adjusted for maternal age at delivery, parity, country of birth, cohabiting status, body mass index during early pregnancy, smoking during pregnancy, diabetic and hypertensive diseases, calendar period of delivery, parental highest educational level, parental history of neurological or psychiatric disorder, and infant’s sex and birth weight for gestational age. Children born at 40^+0^ weeks are the reference. Any impairment was defined by at least one of the following: motor, cognitive, epileptic, visual, or hearing impairment. Any severe or major impairment was defined by a diagnosis of cerebral palsy, severe mental retardation, generalised epileptic disorder, or severe hearing or visual impairment

Overall, compared with children born full term, children born moderately or late preterm showed higher risks for any impairment; motor, cognitive, epileptic, visual, and hearing impairments; and severe or major neurodevelopmental impairment (table 2). For example, the highest relative risk of neurodevelopmental impairment for children born moderately preterm compared with infants born full term was for motor impairment, with a hazard ratio of 4.70 (95% confidence interval 3.95 to 5.59). The risk difference for any impairment was 4.75% (95% confidence interval 3.88% to 5.60%)—that is, 475 (95% confidence interval 388 to 560) cases per 10 000 population by age 16 years, when comparing children born moderately preterm with those born full term, showing the highest absolute risk of neurodevelopmental impairment. Children born early term also showed higher risks of neurodevelopmental impairments than children born full term (table 2). When neurodevelopmental outcomes were assessed by gestational age as a continuum, the risks (both relative (hazard ratio) and absolute (risk difference)) for neurodevelopmental impairments were highest at 32^+0^ gestational weeks, then gradually declined until 41^+6^ weeks (fig 1 and supplementary table D). Population attributable fractions corresponding to changes in gestational age group showed that the greatest reduction in absolute risk for any neurodevelopmental impairment would be seen in children born at 37-38 weeks if they were born later at 39-40 weeks (2.24%, 95% confidence interval 1.71% to 2.76%). For severe or major impairment, the highest population attributable fractions were observed for children born moderately or late preterm (see supplementary table E). Among children born preterm, birth weight for gestational age between the third and 10th centile was associated with higher risks of any impairment, as well as motor, cognitive, and hearing impairment; these risks, plus those of epileptic, visual, and severe or major impairments, were highest in the lowest birth weight for gestational age (<3rd centile) category (table 3).

**Table 2:** Neurodevelopmental outcomes by gestational age (32-41 weeks) among liveborn singleton children without congenital malformations in Sweden 1998-2012

Gestational age (weeks)	Composite outcome*	Neurodevelopmental impairment					
		Motor	Cognitive	Epileptic	Visual	Hearing	Severe or major†
**Moderately preterm: 32-33 (n=7525)**							
Person years	90 313	94 591	94 474	95 477	95 059	95 273	94 761
No with outcome (rate‡)	833 (92.2)	205 (21.7)	335 (35.5)	146 (15.3)	202 (21.2)	193 (20.3)	198 (20.9)
Hazard ratio (95% CI)§	1.73 (1.60 to 1.87)	4.70 (3.95 to 5.59)	1.74 (1.54 to 1.97)	1.92 (1.59 to 2.31)	1.72 (1.47 to 2.01)	1.39 (1.18 to 1.64)	3.56 (3.00 to 4.22)
Risk difference (%) (95% CI)§¶	4.75 (3.88 to 5.60)	1.66 (1.31 to 1.97)	2.02 (1.56 to 2.51)	0.97 (0.59 to 1.41)	1.24 (0.74 to 1.65)	0.71 (0.35 to 1.13)	1.76 (1.42 to 2.06)
**Late preterm: 34-36 (n=48 772)**							
Person years	598 343	621 584	616 565	621 077	618 083	618 996	621 694
No with outcome (rate‡)	3882 (64.9)	439 (7.1)	1492 (24.2)	592 (9.5)	1082 (17.5)	953 (15.4)	495 (8.0)
Hazard ratio (95% CI)§	1.30 (1.26 to 1.35)	1.90 (1.70 to 2.13)	1.31 (1.24 to 1.39)	1.23 (1.12 to 1.36)	1.42 (1.32 to 1.52)	1.16 (1.08 to 1.25)	1.55 (1.40 to 1.72)
Risk difference (%) (95% CI)§¶	2.03 (1.75 to 2.35)	0.40 (0.32 to 0.50)	0.88 (0.72 to 1.10)	0.25 (0.13 to 0.36)	0.71 (0.58 to 0.89)	0.29 (0.12 to 0.42)	0.37 (0.25 to 0.48)
**Early term: 37-38 (n=257 591)**							
Person years	3 169 387	3 266 114	3 241 587	3 260 377	3 249 580	3 248 389	3 265 620
No with outcome (rate‡)	16 269 (51.3)	1386 (4.2)	6230 (19.2)	2468 (7.6)	4244 (13.1)	4302 (13.2)	1671 (5.1)
Hazard ratio (95% CI)§	1.08 (1.06 to 1.11)	1.28 (1.20 to 1.38)	1.14 (1.10 to 1.17)	1.06 (1.01 to 1.11)	1.10 (1.05 to 1.14)	1.04 (1.00 to 1.08)	1.10 (1.03 to 1.17)
Risk difference (%) (95% CI)§¶	0.57 (0.42 to 0.71)	0.13 (0.08 to 0.16)	0.38 (0.29 to 0.48)	0.06 (−0.00 to 0.12)	0.17 (0.10 to 0.23)	0.08 (0.00 to 0.16)	0.06 (0.02 to 0.11)
**Full term: 39-40 (n=713 952)**							
Person years	8 776 743	9 016 313	8 957 542	8 994 774	8 972 130	8 965 555	9 010 016
No with outcome (rate‡)	40 114 (45.7)	2845 (3.2)	14 278 (15.9)	6415 (7.1)	10 419 (11.6)	11 064 (12.3)	4154 (4.6)
Hazard ratio (95% CI)§	Reference	Reference	Reference	Reference	Reference	Reference	Reference
Risk difference (%) (95% CI)§¶	Reference	Reference	Reference	Reference	Reference	Reference	Reference
**Late term: 41 (n=253 850)**							
Person years	3 137 692	3 223 216	3 202 278	3 215 850	3 206 799	3 205 576	3 220 728
No with outcome (rate‡)	14 213 (45.3)	1024 (3.2)	5036 (15.7)	2249 (7.0)	3753 (11.7)	3881 (12.1)	1534 (4.8)
Hazard ratio (95% CI)§	0.98 (0.96 to 1.00)	0.95 (0.88 to 1.03)	0.97 (0.93 to 1.00)	0.96 (0.91 to 1.01)	1.01 (0.97 to 1.05)	0.98 (0.94 to 1.02)	1.01 (0.95 to 1.07)
Risk difference (%) (95% CI)§¶	−0.12 (−0.24 to 0.01)	−0.02 (−0.05 to 0.02)	−0.09 (−0.16 to −0.01)	−0.04 (−0.09 to 0.02)	0.02 (−0.07 to 0.10)	−0.04 (−0.10 to 0.02)	0.00 (−0.03 to 0.05)

CI=confidence interval.

* At least one of motor, cognitive, epileptic, visual, or hearing impairment.

† Diagnosis of cerebral palsy, severe mental retardation, generalised epileptic disorder, or severe hearing or visual impairment.

‡ Number with outcome per 10 000 person years.

§ Adjusted for maternal age at delivery, parity, country of birth, cohabiting status, body mass index during early pregnancy, smoking during pregnancy, diabetic and hypertensive diseases, calendar period of delivery, parental highest educational level, parental history of neurological or psychiatric disorder, infant’s sex, and birth weight for gestational age.

¶ Difference in risk of a specific neurodevelopmental outcome by age 16 years comparing different gestational age groups.

**Table 3:** Neurodevelopmental outcomes by birth weight for gestational age among preterm (32-36 weeks) liveborn singleton children without congenital malformations in Sweden 1998-2012 (n=55 746)

Birth weight for gestational age (centiles)	Composite outcome*	Neurodevelopmental impairment					
		Motor	Cognitive	Epileptic	Visual	Hearing	Severe or major†
**<3rd (n=4027)**							
Person years	47 369	50 391	49 865	50 635	50 307	50 394	50 439
No with outcome (rate‡)	524 (110.6)	95 (18.9)	233 (46.7)	81 (16.0)	133 (26.4)	129 (25.6)	100 (19.8)
Hazard ratio (95% CI)§	1.65 (1.47 to 1.85)	2.27 (1.72 to 3.00)	1.83 (1.53 to 2.18)	1.78 (1.35 to 2.37)	1.50 (1.20 to 1.87)	1.66 (1.30 to 2.11)	2.27 (1.74 to 2.96)
**3rd-10th (n=4346)**							
Person years	52 572	54 860	54 447	55 000	54 738	54 688	54 981
No with outcome (rate‡)	419 (79.7)	63 (11.5)	170 (31.2)	63 (11.5)	107 (19.5)	112 (20.5)	55 (10.0)
Hazard ratio (95% CI)§	1.24 (1.11 to 1.39)	1.39 (1.03 to 1.88)	1.32 (1.10 to 1.59)	1.24 (0.92 to 1.68)	1.08 (0.86 to 1.36)	1.43 (1.14 to 1.79)	1.18 (0.86 to 1.62)
**10th-90th (n=40 988)**							
Person years	501 082	519 774	516 333	519 725	517 296	518 269	519 835
No with outcome (rate‡)	3187 (63.6)	409 (7.9)	1191 (23.1)	501 (9.6)	887 (17.1)	756 (14.6)	454 (8.7)
Hazard ratio (95% CI)§	Reference	Reference	Reference	Reference	Reference	Reference	Reference
**90th-97th (n=3219)**							
Person years	40 817	42 295	41 961	42 270	42 111	42 157	42 320
No with outcome (rate‡)	240 (58.8)	31 (7.3)	90 (21.4)	38 (9.0)	66 (15.7)	63 (14.9)	30 (7.1)
Hazard ratio (95% CI)§	0.86 (0.74 to 1.00)	0.86 (0.57 to 1.30)	0.83 (0.65 to 1.05)	0.82 (0.56 to 1.20)	0.90 (0.68 to 1.19)	0.96 (0.71 to 1.28)	0.81 (0.54 to 1.22)
**≥97th (n=3166)**							
Person years	39 718	41 419	41 010	41 459	41 299	41 307	41 438
No with outcome (rate‡)	284 (71.5)	35 (8.5)	122 (29.7)	44 (10.6)	72 (17.4)	69 (16.7)	41 (9.9)
Hazard ratio (95% CI)§	1.01 (0.88 to 1.17)	0.71 (0.47 to 1.08)	1.07 (0.86 to 1.33)	0.89 (0.61 to 1.29)	1.06 (0.81 to 1.37)	1.13 (0.85 to 1.50)	0.79 (0.53 to 1.20)

CI=confidence interval.

* At least one of motor, cognitive, epileptic, visual, or hearing impairment.

† Diagnosis of cerebral palsy, severe mental retardation, generalised epileptic disorder, or severe hearing or visual impairment.

‡ Number with outcome per 10 000 person years.

§ Adjusted for maternal age at delivery, parity, country of birth, cohabiting status, body mass index during early pregnancy, smoking during pregnancy, diabetic and hypertensive diseases, calendar period of delivery, parental highest educational level, parental history of neurological or psychiatric disorder, infant's sex, and gestational age.

After multiple imputations of missing data, the association between gestational age and neurodevelopmental impairment was largely unchanged (see supplementary table F). A comparison analysis on a subset of 349 108 full siblings showed similar results except that no evidence was observed for associations between gestational age and epileptic or hearing impairment; children born early term had a higher risk for cognitive impairment only, compared with children born full term (see supplementary table G). After stratifying on onset of labour, we observed overall similar risk patterns between spontaneous and induced labour, with some higher risks for motor and severe or major impairment for children born spontaneously at 32-33 weeks, and for any and cognitive impairment for children born spontaneously at 37-38 weeks, compared with their counterparts born through induced labour (see supplementary table H). Similar results were observed when considering only children born from 2001 to 2012 (see supplementary table I).

## Discussion

In this Swedish nationwide cohort study of more than one million children born at 32-41 weeks, we found those born moderately preterm (32-33 weeks) or late preterm (34-36 weeks) showed higher risks of any long term neurodevelopmental outcome, such as motor, cognitive, and visual impairment, than children born full term (39-40 weeks). These risks were highest at the earliest gestational age (from 32 weeks), and gradually decreased as gestational age increased, with higher risks also at early term (37-38 weeks) than at full term. Among children born preterm, those born small for gestational age, especially in the <3rd centile, showed higher risks of long term neurodevelopmental impairment than those born preterm with normal birth weight for gestational age.

### Strengths and limitations of this study

A major strength of the study is the population based design and the large sample size using comprehensive national registries with high validity, making it possible to investigate clinically relevant risks across the spectrum of gestational age. This study provided a detailed overview of long term neurodevelopmental outcomes among infants born at 32-41 gestational weeks from a nationwide cohort. As children born moderately or late preterm receive the same routine care as children born at term in Sweden as in many other countries,^52^ ^53^ misclassification of outcomes related to gestational age is unlikely. We were able to adjust for potential confounders known to affect both gestational age and neurodevelopment, based on prospectively collected data on gestational age, covariates, and outcomes from the first visit to antenatal care to discharge from delivery hospital, as well as inpatient and outpatient care. Apart from hazard ratios, we also estimated risk differences and population attributable fractions to provide a comprehensive picture of the studied associations and the public health impact of preterm birth.

This study has also some limitations. We were unable to provide precise information on neurodevelopmental outcomes, such as intelligence quotient, owing to the non-granular nature of the data. Some neurodevelopmental outcomes such as autism spectrum disorders and attention deficit/hyperactivity disorder were not included, and it was not possible to distinguish between types or severity of some of the impairments owing to an overlap in clinical signs. This might have led to the outcome diagnoses being underreported or misclassified, which could result in an underestimation of associations. Competing risk of death might be present but its possible impact on the estimated associations is considered negligible because death is a rare event in this study population. Coverage of data from public inpatient and outpatient care is almost 100%, but coverage of data from private specialised care is estimated to be lower, even if it is mandatory for all public and private care providers to deliver data to the Patient Register.^54^ This could result in the number of affected children being underreported. Unmeasured confounding, such as alcohol and substance misuse during pregnancy, and treatment with antenatal steroids before preterm delivery, might have influenced our results. Moreover, given the observational nature of the study, we cannot draw conclusions about the causal relationship between gestational age and neurodevelopmental impairment. Lastly, despite adjusting for calendar period of delivery, developments in obstetric and neonatal care may have influenced the association between gestational age and outcomes over the 15 years of the study period.

### Comparison with other studies

Our findings confirm and expand on the results of earlier studies describing higher risks of adverse neurodevelopmental outcomes among children born moderately or late preterm.^8^ ^9^ ^10^ ^11^ ^12^ ^13^ ^14^ ^15^ ^17^ ^18^ ^19^ ^20^ ^21^ ^22^ ^23^ ^24^ ^25^ ^55^ Comparisons of long term outcomes for those children is challenging as most published studies only evaluated outcomes at 2 years or 36 months of age,^8^ ^9^ ^10^ ^11^ ^18^ ^20^ ^21^ or evaluated different outcomes, such as school performance.^22^ ^23^ ^24^ Nevertheless, the prevalence of motor, visual, and hearing impairment for infants born at 32-34 weeks in our study are in line with those reported from the EPIPAGE-2 (an epidemiological study on small gestational ages) cohort study,^15^ even if the exact definitions of outcomes and lengths of follow-up were not similar. Moreover, we described in detail associations between gestational week and risks of different outcomes with long term follow-up. Interestingly, not only children born moderately or late preterm but also those born early term faced higher risks of adverse neurodevelopmental outcomes. When looking at the whole spectrum of term gestation, children born early term have been reported to have higher risks compared with children born full term for neonatal morbidities during the neonatal period,^7^ and for motor and cognitive impairments and lower academic performance during early childhood. 14 22 56 In the sibling comparison analysis, the associations between early term birth and neurodevelopmental impairments were attenuated to null. This suggests that the associations between early term birth and adverse neurodevelopmental outcomes might be explained by shared genetic and environmental factors. However, null findings may also imply that the impact of early term birth on neurodevelopment mediated only through familial factors is “controlled away” in sibling comparison analysis.^57^ Moreover, given that the subset of full siblings only accounts for about a quarter of the entire population, this result might be prone to type II error and should be interpreted with caution.

Weekly increased risks have already been reported for autism spectrum disorder by decreasing gestational weeks, in children born full term to early term and to preterm in Sweden.^38^ ^39^ All these increased risks have an adverse impact on early school performance,^13^ ^21^ ^22^ ^23^ income, and possibilities of completing a university education.^58^ Although absolute risks are low, even small shifts in the gestational age spectrum might have implications for public health, as moderately or late preterm births constitute 84% of preterm births in Sweden and nearly 80% of preterm births in other high income countries.^41^ ^59^

### Implications and future work

Compared with children born extremely or very preterm, those born moderately or late preterm are considered as low risk, and in many countries are not included in follow-up programmes.^52^ However, our results support the findings of no clear cut-off limit before 40 gestational weeks when children can be considered as fully mature,^7^ ^60^ ^61^ ^62^ as children born moderately or late preterm and also early term are more vulnerable compared with children born full term. Results on low absolute risks may help professionals when advising parents and families about risk, to avoid unnecessary anxiety and reassure them. Our findings may also help obstetricians and neonatologists balance the advantages and disadvantages of induced labour in cases of non-spontaneous birth. Professionals must be aware that it might be possible to lower risks in children born preterm or early term by delaying birth and restricting induction of labour before 39 weeks, except for medical reasons.^63^ During follow-up of this large population of children born preterm, primary care practitioners, general practitioners, and paediatricians need to be aware of the difficulties that families might face, and be alert to parental concerns to avoid delayed referrals to specialised services for these children, particularly for those born preterm and small for gestational age. Our findings support the strategy to prevent births before full term to decrease the risk of neurodevelopmental impairments. Targeting health policies focused on population risk factors for the full spectrum of early delivery (<39 weeks), including pregnancy complications, maternal sociodemographic and lifestyle characteristics, environmental factors, and medical practices (eg, provider initiated delivery) could have a synergistic impact on the avoidance of early delivery.^41^ Future studies could evaluate causal pathways resulting in adverse outcomes, such as the reason for prematurity and neonatal morbidities,^64^ and strategies for prevention or intervention. It might also be considered whether a larger proportion of children born preterm should be subjected to some structural follow-up after discharge from neonatal care, especially those born small for gestational age. Also, improving the knowledge of education professionals about the needs of children born preterm might improve early recognition and referral to specialised services and thus enhance appropriate support for these children.^15^

### Conclusion

In this large population based cohort study, we found long term neurodevelopmental impairments in a broad range of areas among the largest group of children born preterm, reflecting the continuity of risk across the gestational age spectrum. This global perspective is important when advising parents and health professionals, and also when planning healthcare systems for children born preterm. Our findings support that preventing moderately or late preterm delivery may have implications for public health, and that higher risks faced by these groups of children and their families should not be underestimated.

**What is already known on this topic**

Children born moderately preterm (32-33 weeks) or late preterm (34-36 weeks) represent a substantial healthcare burden in neonatal medicine
Although reports suggest higher risks of neurodevelopmental impairments in children born moderately or late preterm, few population based studies have investigated the long term neurodevelopmental outcomes of these children compared with children born at term

**What this study adds**

In liveborn singleton children without congenital malformations, risks for neurodevelopmental impairments were highest at 32 gestational weeks, and gradually decreased until 41 weeks
Even small absolute risks should not be underestimated as these preterm children comprise the largest proportion of children born preterm
The findings may help professionals and families to better assess risk, follow-up, and healthcare systems planning for children born moderately or late preterm

## Data Availability

No additional data available.

## Web extra

Extra material supplied by authors

## Notes

Contributors: AM, RC, and JB conceived and designed the study. AM and RC analysed the data. AM wrote the first draft of the manuscript. All authors contributed to the writing of the manuscript, interpreted the data, critically revised the manuscript for important intellectual content, and agreed to be accountable for all aspects of the work. OS, NR, and JB obtained funding. NR and OS provided administrative, technical, and material support. All authors had full access to all the data in the study and take full responsibility for the integrity of the data and the accuracy of the data analysis. They are the guarantors. The corresponding author attests that all listed authors meet authorship criteria and that no others meeting the criteria have been omitted.

The lead authors (the manuscript’s guarantors) affirms that the manuscript is an honest, accurate, and transparent account of the study being reported; that no important aspects of the study have been omitted; and that any discrepancies from the study as planned (and, if relevant, registered) have been explained.

Dissemination to participants and related patient and public communities: The findings of this study will be disseminated through the media departments and websites of the authors’ institutes, and through press releases and social media.

Provenance and peer review: Not commissioned; externally peer reviewed.
