# Supplementary material for: Neurological development in children born moderately or late preterm: national cohort study
Source: BMJ. 2024 Jan 24;384:e075630. doi: 10.1136/bmj-2023-075630 (PMC11957549; doi:10.1136/bmj-2023-075630)
Supplement: Supplementary file 1 — Supplementary information: Additional figures A and B and tables A-I [file mita075630.ww.pdf]

Supplementary material

Supplementary Figure A. Flow chart of inclusion and exclusion of the study population.

Supplementary Figure B. Directed acyclic graph illustrating possible structural relationship between gestational age, confounders, and neurodevelopmental impairments.

Supplementary Table A. ICD-10 codes for parental and infant diseases and outcomes.

Supplementary Table B. Diagnosis source for the first diagnosis of each neurodevelopmental outcome.

Supplementary Table C. Number of neurodevelopmental outcomes impaired among singleton live-born moderately and late preterm born children and term children without congenital malformations in Sweden 1998-2012.

Supplementary Table D. Association between each completed week of gestational age from 32 to 41 week and neurodevelopmental outcomes among singleton live-born children without congenital malformations in Sweden 1998-2012.

Supplementary Table E. Population attributable fraction for neurodevelopmental outcomes across gestational age (32 to 41 weeks) among singleton live-born children without congenital malformations in Sweden 1998-2012.

Supplementary Table F. Association between gestational age (32 to 41 weeks) and neurodevelopmental outcomes among singleton live-born children without congenital malformations in Sweden 1998-2012 (multiple imputation analysis).

Supplementary Table G. Association between gestational age (32 to 41 weeks) and neurodevelopmental outcomes among full sibling of singleton live-born children without congenital malformations in Sweden 1998-2012 (sibling comparison analysis).

Supplementary Table H. Association between gestational age (32 to 41 weeks) and neurodevelopmental outcomes among singleton live-born children without congenital malformations in Sweden 1998-2012, stratified by onset of labor.

Supplementary Table I. Association between gestational age (32 to 41 weeks) and neurodevelopmental outcomes among singleton live-born children without congenital malformations in Sweden 2001-2012.

Supplementary Figure A. Flow chart of inclusion and exclusion of the study population.

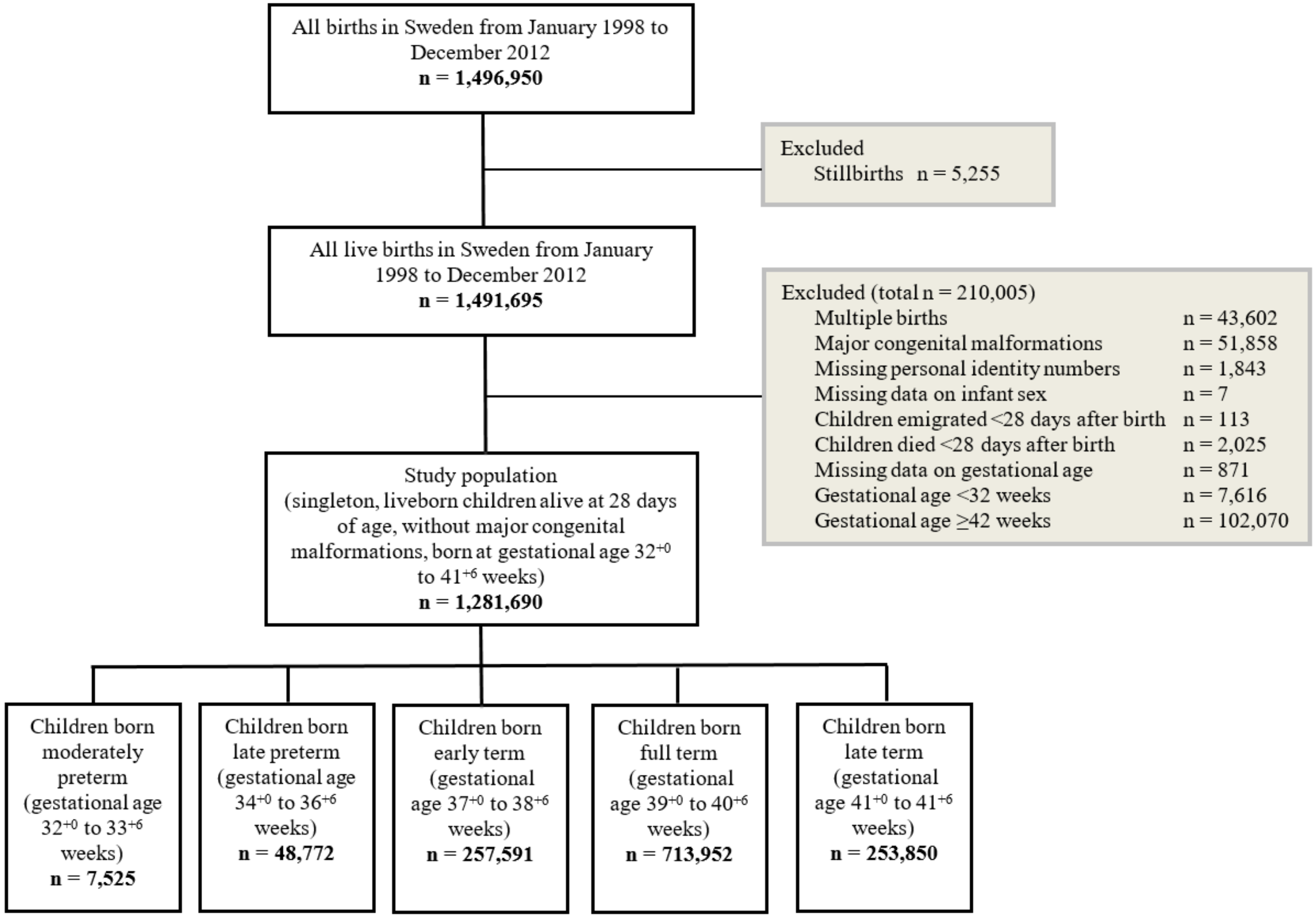

Supplementary Figure B. Directed acyclic graph illustrating possible structural relationship between gestational age, confounders, and neurodevelopmental impairments.

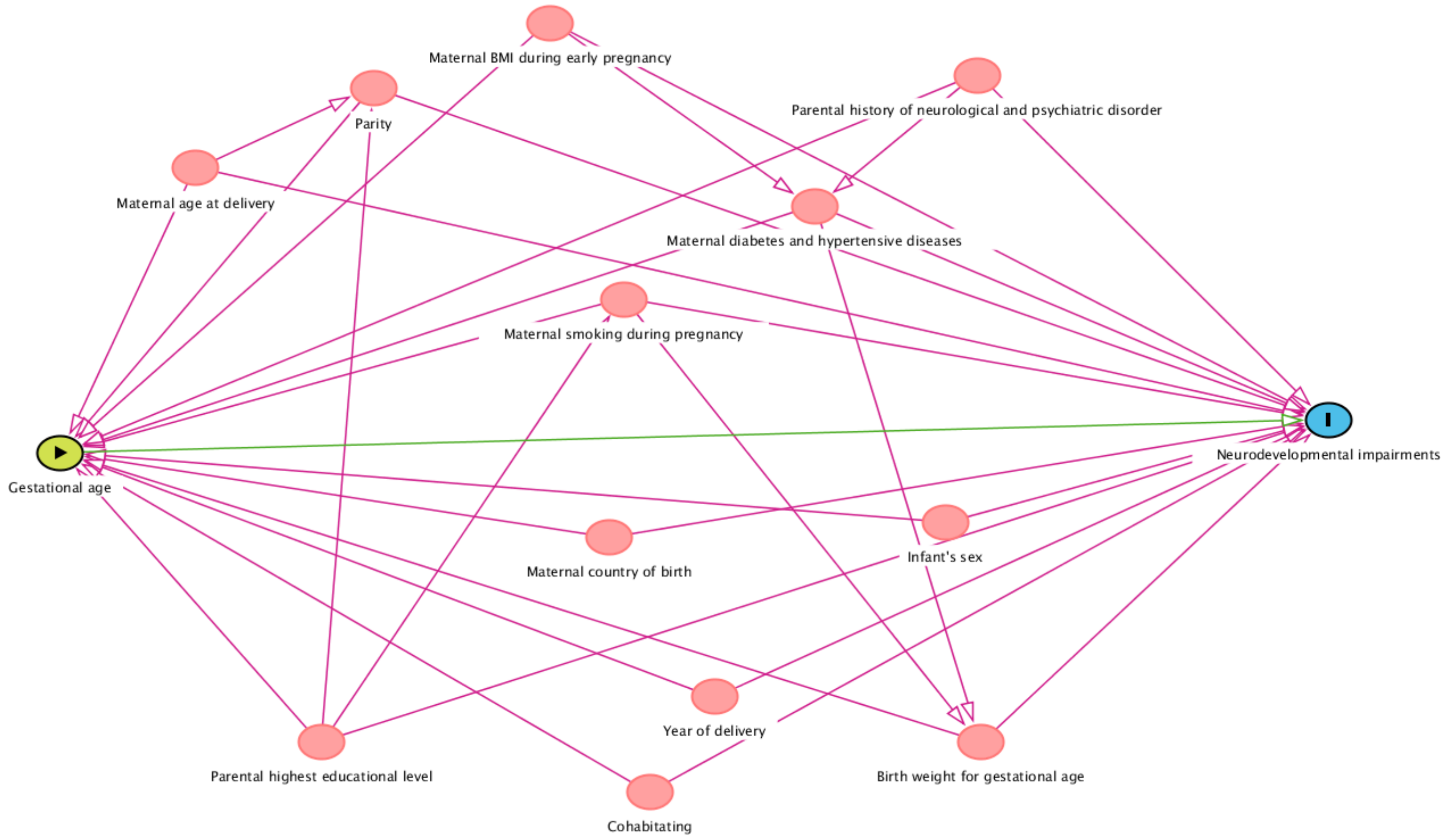

Supplementary Table A. ICD-10 codes for parental and infant diseases and outcomes

Diagnoses	ICD-10 codes
Major congenital malformation	Q00-Q99, excluding minor malformations[a]: Q17.0 (accessory auricle), Q17.5 (prominent ear), Q18.0/Q18.1 (branchial cleft or preaurical sinus), Q25.0 (patent ductus arterious), Q27.0 (absence of umbilical artery), Q31.4/Q32.0 (laryngomalacia), Q38.1 (anchyloglossia), Q52.3 (imperforate hymen), Q53.0-.9 (retentio testis), Q65.0-.9 (congenital hip dislocation), Q66.5-.9 (pes planus), Q69.0/Q69.9 (polydactali), Q70.3 (syndactyly), Q76.0 (spina bifida occulta), Q79.9 (unspecified anomalies of the musculoskeletal system), Q82.5/Q82.9 (congenital nevus)
Parental history of neurological or psychiatric disorders	F00-F99 (Mental and behavioural disorders)<br>G00-G99 (Diseases of the nervous system)<br>290-319[b] (Mental disorders)<br>320-326[b] (Inflammatory diseases of the central nervous system)<br>330-337[b] (Hereditary and Degenerative diseases of the central nervous system)<br>340-359[b] (Other disorders of the central nervous system)<br>435-436[b] (Transient cerebral ischemia and Acute, but ill-defined, cerebrovascular disease)
Maternal diabetes	E10-E14, and O240-O243 (Pre-gestational diabetes)<br>O244 (Gestational diabetes)
Maternal hypertensive diseases	I10-I15, O10, and O11 (Chronic hypertension)<br>O14 and O15 (Preeclampsia)
Motor impairment	G80 (Cerebral palsy) and F82 (Specific developmental disorder of motor function)
Cognitive impairment	F70, F71, F72, F73, F78, F79 (Mental retardation)<br>F80.0, F80.1, F80.2, F80.8, F80.9 (Specific developmental disorders of speech and language)<br>F81.0, F81.1, F81.2, F81.3, F81.8, F81.9 (Specific developmental disorders of scholastic skills)<br>F83 (Mixed specific developmental disorders)
Epileptic impairment	G40 (Status epilepticus)<br>G41 (Epilepsy and recurrent seizures)
Visual impairment	H53, H54 (Visual disturbances and blindness)
Hearing impairment	H90, H91 (Hearing loss)
Severe or major impairment	G80 (Cerebral palsy)<br>F72, F73 (Severe or profound mental retardation)<br>G41 (Status epilepticus), G40.3 (Generalised idiopathic epilepsy and epileptic syndromes), G40.6 (Grand mal seizures, unspecified (with or without petit mal)), G40.7 (Petit mal, unspecified, without grand mal seizures)

	H90.0 (Conductive hearing loss bilateral)<br>H54.0, H54.1 (Blindness binocular or Severe visual impairment binocular)

[a] The excluded diagnoses are minor malformations, defined by the Swedish National Board of Health and Welfare (see https://www.socialstyrelsen.se/globalassets/sharepoint-dokument/dokument-webb/ovrigt/diagnoser-som-inte-ska-rapporteras-om-fosterskador.pdf).

[b] ICD-9 codes for the diagnoses during the years 1987-1998.

Supplementary Table B. Diagnosis source for the first diagnosis of each neurodevelopmental outcome

Diagnosis source	Any impairment[a]	Any motor impairment	Any cognitive impairment	Any epileptic impairment	Any visual impairment	Any hearing impairment	Any severe or major impairment[b]
Total (N[%])	75,311 (100.0)	5,899 (100.0)	27,371 (100.0)	11,870 (100.0)	19,700 (100.0)	20,393 (100.0)	8,052 (100.0)
Hospitalized care (N[%])	3,619 (4.8)	321 (5.4)	467 (1.7)	3,196 (26.9)	318 (1.6)	182 (0.9)	1,087 (13.5)
Specialized outpatient care (N[%])	71,692 (95.2)	5,578 (94.6)	26,904 (98.3)	8,674 (73.1)	19,382 (98.4)	20,211 (99.1)	6,965 (86.5)

[a]At least one of motor, cognitive, epileptic, visual, or hearing impairment.

[b]Diagnosis of cerebral palsy, severe mental retardation, generalised epileptic disorder, or severe hearing or visual impairment.

Supplementary Table C. Number of neurodevelopmental outcomes impaired among singleton non-malformed live-born moderate and late preterm infants and term infants in Sweden 1998-2012

**Number of outcomes[a] impaired**	**Gestational age (weeks; N(%))**				
	**32-33**	**34-36**	**37-38**	**39-40**	**41**
Total	7,525 (100.0)	48,772 (100.0)	257,591 (100.0)	713,952 (100.0)	253,850 (100.0)
0	6,692 (88.9)	44,890 (92.0)	241,322 (93.7)	673,838 (94.4)	239,637 (94.4)
1	660 (8.8)	3,368 (6.9)	14,352 (5.6)	36,172 (5.1)	12,830 (5.1)
2	117 (1.6)	388 (0.8)	1,552 (0.6)	3,155 (0.4)	1,102 (0.4)
3	38 (0.5)	91 (0.2)	290 (0.1)	623 (0.1)	220 (0.1)
4	17 (0.2)	34 (0.1)	71 (0.0)	150 (0.0)	56 (0.0)
5	1 (0.0)	1 (0.0)	4 (0.0)	14 (0.0)	5 (0.0)

[a] Motor or/and cognitive or/and epileptic or/and hearing or/and visual impairment.

Supplementary Table D. Association between each completed week of gestational age from 32 to 41 weeks and neurodevelopmental outcomes among singleton live-born children without congenital malformations in Sweden 1998-2012

Gestational age (weeks)	Any impairment[a]	Any motor impairment	Any cognitive impairment	Any epileptic impairment	Any visual impairment	Any hearing impairment	Any severe or major impairment[b]
32 (n=2,994)							
Person-years	35,371	37,130	37,275	37,713	37,499	37,714	37,228
No. of cases (Rate[c])	362 (102.3)	110 (29.6)	136 (36.5)	68 (18.0)	94 (25.1)	71 (18.8)	103 (27.7)
HR (95% CI)[d]	1.92 (1.70 to 2.17)	6.74 (5.35 to 8.49)	1.69 (1.39 to 2.07)	2.32 (1.77 to 3.04)	2.09 (1.67 to 2.62)	1.25 (0.95 to 1.64)	4.77 (3.78 to 6.01)
RD (%) (95% CI)[d,e]	5.93 (4.80 to 7.13)	2.46 (1.87 to 3.11)	1.85 (1.05 to 2.61)	1.38 (0.73 to 2.10)	1.91 (1.06 to 2.56)	0.47 (-0.06 to 1.12)	2.56 (1.83 to 3.27)
33 (n=4,531)							
Person-years	54,942	57,462	57,199	57,765	57,561	57,559	57,532
No. of cases (Rate[c])	471 (85.7)	95 (16.5)	199 (34.8)	78 (13.5)	108 (18.8)	122 (21.2)	95 (16.5)
HR (95% CI)[d]	1.64 (1.48 to 1.82)	3.71 (2.89 to 4.75)	1.85 (1.58 to 2.16)	1.70 (1.32 to 2.20)	1.50 (1.21 to 1.87)	1.51 (1.23 to 1.85)	2.86 (2.25 to 3.64)
RD (%) (95% CI)[d,e]	4.12 (3.02 to 4.99)	1.16 (0.77 to 1.45)	2.24 (1.65 to 2.89)	0.74 (0.27 to 1.27)	0.84 (0.23 to 1.38)	0.90 (0.34 to 1.52)	1.26 (0.78 to 1.65)
34 (n=7,993)							
Person-years	97,710	101,779	101,242	101,858	101,293	101,352	101,698
No. of cases (Rate[c])	689 (70.5)	97 (9.5)	242 (23.9)	101 (9.9)	203 (20.0)	182 (18.0)	116 (11.4)
HR (95% CI)[d]	1.41 (1.30 to 1.54)	2.47 (1.96 to 3.11)	1.29 (1.12 to 1.48)	1.37 (1.10 to 1.69)	1.55 (1.32 to 1.82)	1.38 (1.18 to 1.63)	2.26 (1.84 to 2.78)
RD (%) (95% CI)[d,e]	2.70 (2.03 to 3.64)	0.64 (0.46 to 0.89)	0.79 (0.26 to 1.34)	0.38 (0.15 to 0.63)	0.92 (0.56 to 1.33)	0.67 (0.20 to 1.13)	0.85 (0.57 to 1.09)
35 (n=13,634)							
Person-years	166,787	173,464	172,144	173,391	172,418	172,791	173,529
No. of cases (Rate[c])	1,094 (65.6)	136 (7.8)	419 (24.3)	164 (9.5)	323 (18.7)	270 (15.6)	147 (8.5)
HR (95% CI)[d]	1.35 (1.27 to 1.45)	2.26 (1.87 to 2.73)	1.36 (1.22 to 1.52)	1.19 (1.00 to 1.43)	1.56 (1.38 to 1.76)	1.24 (1.09 to 1.42)	1.65 (1.36 to 1.98)
RD (%) (95% CI)[d,e]	2.34 (1.74 to 2.87)	0.55 (0.40 to 0.73)	0.98 (0.66 to 1.43)	0.21 (-0.05 to 0.44)	0.95 (0.69 to 1.28)	0.43 (0.15 to 0.68)	0.44 (0.23 to 0.64)
36 (n=27,145)							
Person-years	333,846	346,342	343,179	345,828	344,373	344,853	346,467
No. of cases (Rate[c])	2,099 (62.9)	206 (5.9)	831 (24.2)	327 (9.5)	556 (16.1)	501 (14.5)	232 (6.7)

HR (95% CI)[d]	1.27 (1.21 to 1.34)	1.70 (1.45 to 2.00)	1.35 (1.25 to 1.46)	1.25 (1.10 to 1.42)	1.34 (1.22 to 1.48)	1.08 (0.97 to 1.19)	1.33 (1.15 to 1.55)
RD (%) (95% CI)[d,e]	1.83 (1.49 to 2.29)	0.30 (0.19 to 0.39)	0.97 (0.74 to 1.25)	0.27 (0.14 to 0.41)	0.57 (0.40 to 0.79)	0.15 (-0.08 to 0.36)	0.22 (0.09 to 0.31)
37 (n=65,921)							
Person-years	811,872	838,129	831,290	836,671	834,140	833,648	837,973
No. of cases (Rate[c])	4,415 (54.4)	412 (4.9)	1,749 (21.0)	685 (8.2)	1,131 (13.6)	1,135 (13.6)	485 (5.8)
HR (95% CI)[d]	1.13 (1.09 to 1.17)	1.48 (1.31 to 1.66)	1.22 (1.15 to 1.29)	1.14 (1.05 to 1.25)	1.12 (1.05 to 1.20)	1.06 (0.98 to 1.13)	1.21 (1.09 to 1.34)
RD (%) (95% CI)[d,e]	0.87 (0.60 to 1.07)	0.21 (0.13 to 0.28)	0.59 (0.45 to 0.74)	0.15 (0.03 to 0.26)	0.21 (0.08 to 0.33)	0.10 (-0.04 to 0.23)	0.14 (0.08 to 0.21)
38 (n=191,670)							
Person-years	2,357,515	2,427,986	2,410,297	2,423,706	2,415,441	2,414,740	2,427,648
No. of cases (Rate[c])	11,854 (50.3)	974 (4.0)	4,481 (18.6)	1,783 (7.4)	3,113 (12.9)	3,167 (13.1)	1,186 (4.9)
HR (95% CI)[d]	1.09 (1.06 to 1.11)	1.29 (1.18 to 1.41)	1.14 (1.10 to 1.19)	1.05 (0.99 to 1.12)	1.10 (1.05 to 1.16)	1.05 (1.01 to 1.10)	1.08 (1.00 to 1.16)
RD (%) (95% CI)[d,e]	0.58 (0.42 to 0.76)	0.12 (0.08 to 0.16)	0.38 (0.27 to 0.51)	0.06 (-0.01 to 0.14)	0.18 (0.10 to 0.26)	0.10 (0.02 to 0.20)	0.05 (-0.00 to 0.11)
39 (n=320,882)							
Person-years	3,929,742	4,039,095	4,012,059	4,029,410	4,019,430	4,016,386	4,036,364
No. of cases (Rate[c])	18,270 (46.5)	1,335 (3.3)	6,604 (16.5)	2,933 (7.3)	4,718 (11.7)	5,047 (12.6)	1,910 (4.7)
HR (95% CI)[d]	1.03 (1.00 to 1.05)	1.10 (1.01 to 1.19)	1.05 (1.01 to 1.09)	1.03 (0.98 to 1.09)	1.02 (0.98 to 1.07)	1.02 (0.98 to 1.07)	1.03 (0.96 to 1.10)
RD (%) (95% CI)[d,e]	0.18 (0.04 to 0.31)	0.04 (0.01 to 0.08)	0.14 (0.05 to 0.22)	0.04 (-0.01 to 0.11)	0.04 (-0.03 to 0.11)	0.04 (-0.01 to 0.11)	0.02 (-0.01 to 0.07)
40 (n=393,070)							
Person-years	4,847,002	4,977,218	4,945,484	4,965,365	4,952,700	4,949,169	4,973,652
No. of cases (Rate[c])	21,844 (45.1)	1,510 (3.0)	7,674 (15.5)	3,482 (7.0)	5,701 (11.5)	6,017 (12.2)	2,244 (4.5)
HR (95% CI)[d]	Reference	Reference	Reference	Reference	Reference	Reference	Reference
RD (%) (95% CI)[d,e]	Reference	Reference	Reference	Reference	Reference	Reference	Reference
41 (n=253,850)							
Person-years	3,137,692	3,223,216	3,202,278	3,215,850	3,206,799	3,205,576	3,220,728
No. of cases (Rate[c])	14,213 (45.3)	1,024 (3.2)	5,036 (15.7)	2,249 (7.0)	3,753 (11.7)	3,881 (12.1)	1,534 (4.8)
HR (95% CI)[d]	0.99 (0.97 to 1.02)	0.99 (0.91 to 1.08)	0.99 (0.95 to 1.03)	0.97 (0.92 to 1.03)	1.02 (0.98 to 1.07)	0.99 (0.95 to 1.03)	1.02 (0.95 to 1.09)
RD (%) (95% CI)[d,e]	-0.04 (-0.17 to 0.10)	-0.00 (-0.03 to 0.04)	-0.02 (-0.11 to 0.05)	-0.02 (-0.09 to 0.05)	0.04 (-0.04 to 0.12)	-0.02 (-0.09 to 0.05)	0.02 (-0.03 to 0.07)

HR=hazard ratio; CI=confidence interval; RD=risk difference.

[a]At least one of motor, cognitive, epileptic, visual, or hearing impairment.

[b]Diagnosis of cerebral palsy, severe mental retardation, generalised epileptic disorder, or severe hearing or visual impairment.

[c]Number with outcome per 10,000 person years.

[d]Adjusted for maternal age at delivery, parity, country of birth, cohabiting status, body mass index during early pregnancy, smoking during pregnancy, diabetic and hypertensive diseases, calendar period of delivery, parental highest educational level, parental history of neurological or psychiatric disorder, infant's sex and birth weight for gestational age.

[e]Difference in risk of a specific neurodevelopmental outcome by 16 years of age comparing different gestational age groups.

Supplementary Table E. Population attributable fraction for neurodevelopmental outcomes across gestational age (32 to 41 weeks) among singleton live-born children without congenital malformations in Sweden 1998-2012

Gestational age (weeks)	Population attributable fraction (%) (95%CI) [a]						
	Any impairment[b]	Any motor impairment	Any cognitive impairment	Any epileptic impairment	Any visual impairment	Any hearing impairment	Any severe or major impairment[c]
32-33	0.78 (0.70 to 0.87)	4.69 (4.47 to 4.91)	0.91 (0.76 to 1.07)	0.99 (0.79 to 1.19)	0.75 (0.59 to 0.92)	0.42 (0.24 to 0.60)	2.92 (2.72 to 3.11)
34-36	1.97 (1.73 to 2.21)	6.18 (5.40 to 6.95)	2.17 (1.75 to 2.58)	1.52 (0.90 to 2.14)	2.65 (2.20 to 3.09)	1.03 (0.55 to 1.51)	3.62 (2.92 to 4.31)
37-38	2.24 (1.71 to 2.76)	7.22 (5.42 to 8.98)	3.59 (2.72 to 4.45)	1.58 (0.24 to 2.90)	2.48 (1.46 to 3.50)	1.13 (0.12 to 2.14)	2.51 (0.88 to 4.11)
39-40	Reference	Reference	Reference	Reference	Reference	Reference	Reference
41	-0.49 (-1.04 to 0.06)	-1.26 (-3.42 to 0.85)	-0.86 (-1.81 to 0.07)	-1.07 (-2.49 to 0.32)	0.33 (-0.73 to 1.38)	-0.56 (-1.61 to 0.48)	0.19 (-1.51 to 1.87)

CI=confidence interval.

[a]Adjusted for maternal age at delivery, parity, country of birth, cohabiting status, body mass index during early pregnancy, smoking during pregnancy, diabetic and hypertensive diseases, calendar period of delivery, parental highest educational level, parental history of neurological or psychiatric disorder, infant's sex and birth weight for gestational age.

[b]At least one of motor, cognitive, epileptic, visual, or hearing impairment.

[c]Diagnosis of cerebral palsy, severe mental retardation, generalised epileptic disorder, or severe hearing or visual impairment.

Supplementary Table F. Association between gestational age (32 to 41 weeks) and neurodevelopmental outcomes among singleton live-born children without congenital malformations in Sweden 1998-2012 (multiple imputation analysis)

Gestational age (weeks)	Hazard ratios (95%CI)[a]						
	Any impairment[b]	Any motor impairment	Any cognitive impairment	Any epileptic impairment	Any visual impairment	Any hearing impairment	Any severe or major impairment[c]
32-33	1.74 (1.62 to 1.87)	4.89 (4.22 to 5.66)	1.72 (1.54 to 1.92)	1.91 (1.61 to 2.25)	1.67 (1.45 to 1.92)	1.47 (1.27 to 1.69)	3.74 (3.23 to 4.32)
34-36	1.31 (1.26 to 1.35)	1.87 (1.69 to 2.07)	1.31 (1.24 to 1.38)	1.25 (1.15 to 1.36)	1.43 (1.35 to 1.53)	1.18 (1.11 to 1.27)	1.57 (1.43 to 1.73)
37-38	1.09 (1.07 to 1.11)	1.27 (1.19 to 1.36)	1.14 (1.10 to 1.17)	1.04 (0.99 to 1.09)	1.10 (1.06 to 1.14)	1.06 (1.02 to 1.09)	1.08 (1.02 to 1.14)
39-40	Reference	Reference	Reference	Reference	Reference	Reference	Reference
41	0.98 (0.97 to 1.00)	0.97 (0.90 to 1.04)	0.97 (0.94 to 1.00)	0.97 (0.93 to 1.02)	1.01 (0.98 to 1.05)	0.98 (0.94 to 1.01)	1.02 (0.97 to 1.09)

CI=confidence interval.

[a]Adjusted for maternal age at delivery, parity, country of birth, cohabiting status, body mass index during early pregnancy, smoking during pregnancy, diabetic and hypertensive diseases, calendar period of delivery, parental highest educational level, parental history of neurological or psychiatric disorder, infant's sex and birth weight for gestational age.

[a]At least one of motor, cognitive, epileptic, visual, or hearing impairment.

[c]Diagnosis of cerebral palsy, severe mental retardation, generalised epileptic disorder, or severe hearing or visual impairment.

Supplementary Table G. Association between gestational age (32 to 41 weeks) and neurodevelopmental outcomes among full sibling of singleton live-born children without congenital malformations in Sweden 1998-2012 (sibling comparison analysis, N=349,108)

Gestational age (weeks)	Any impairment[a]	Any motor impairment	Any cognitive impairment	Any epileptic impairment	Any visual impairment	Any hearing impairment	Any severe or major impairment[b]
32-33							
Person-years	34,199	35,727	35,664	35,973	35,861	35,962	35,721
No. of cases (Rate[c])	277 (81.0)	66 (18.5)	112 (31.4)	52 (14.5)	72 (20.1)	60 (16.7)	73 (20.4)
HR (95% CI)[d]	1.54 (1.27 to 1.87)	3.10 (1.86 to 5.14)	1.46 (1.06 to 2.02)	1.45 (0.93 to 2.29)	1.55 (1.05 to 2.29)	1.13 (0.77 to 1.67)	3.42 (2.11 to 5.54)
34-36							
Person-years	235,222	244,033	242,089	243,956	242,725	242,907	244,126
No. of cases (Rate[c])	1,431 (60.8)	155 (6.4)	543 (22.4)	208 (8.5)	389 (16.0)	367 (15.1)	172 (7.1)
HR (95% CI)[d]	1.21 (1.11 to 1.32)	1.41 (1.07 to 1.86)	1.24 (1.07 to 1.43)	1.04 (0.83 to 1.28)	1.33 (1.13 to 1.57)	1.10 (0.93 to 1.29)	1.30 (1.02 to 1.66)
37-38							
Person-years	1,095,131	1,127,483	1,119,388	1,125,265	1,121,775	1,120,631	1,126,803
No. of cases (Rate[c])	5,337 (48.7)	411 (3.7)	1,985 (17.7)	811 (7.2)	1392 (12.4)	1,488 (13.3)	568 (5.0)
HR (95% CI)[d]	1.04 (0.99 to 1.08)	0.92 (0.79 to 1.07)	1.10 (1.02 to 1.18)	0.90 (0.81 to 1.00)	1.07 (0.99 to 1.16)	1.02 (0.94 to 1.10)	0.95 (0.84 to 1.08)
39-40							
Person-years	1,928,170	1,980,996	1,968,081	1,976,653	1,971,904	1,969,546	1,979,616
No. of cases (Rate[c])	8,785 (45.6)	646 (3.3)	3,090 (15.7)	1,430 (7.2)	2,262 (11.5)	2,509 (12.7)	940 (4.8)
HR (95% CI)[d]	Reference	Reference	Reference	Reference	Reference	Reference	Reference
41							
Person-years	1,054,520	1,083,408	1,076,526	1,080,727	1,077,731	1,076,855	1,082,295
No. of cases (Rate[c])	4,686 (44.4)	311 (2.9)	1,602 (14.9)	755 (7.0)	1,248 (11.6)	1,332 (12.4)	533 (4.9)
HR (95% CI)[d]	1.00 (0.96 to 1.05)	0.91 (0.77 to 1.08)	0.95 (0.87 to 1.02)	1.04 (0.93 to 1.16)	1.03 (0.95 to 1.12)	1.01 (0.93 to 1.09)	1.13 (0.99 to 1.29)

HR=hazard ratio; CI=confidence interval.

[a]At least one of motor, cognitive, epileptic, visual, or hearing impairment.

[b]Diagnosis of cerebral palsy, severe mental retardation, generalised epileptic disorder, or severe hearing or visual impairment.

[c]Number with outcome per 10,000 person years.

[d]Adjusted for maternal age at delivery, parity, cohabiting status, body mass index during early pregnancy, smoking during pregnancy, diabetic and hypertensive diseases, calendar period of delivery, parental history of neurological or psychiatric disorder, infant's sex and birth weight for gestational age.

Supplementary Table H. Association between gestational age (32 to 41 weeks) and neurodevelopmental outcomes among singleton live-born children without congenital malformations in Sweden 1998-2012, stratified by onset of labor (N=1,270,476)

Gestational age (weeks)	Hazard ratios (95%CI)[a]						
	Any impairment[b]	Any motor impairment	Any cognitive impairment	Any epileptic impairment	Any visual impairment	Any hearing impairment	Any severe or major impairment[c]
32-33							
Spontaneous (n=4,720)	1.72 (1.55 to 1.91)	5.93 (4.84 to 7.26)	1.77 (1.51 to 2.09)	1.83 (1.43 to 2.34)	1.62 (1.31 to 1.99)	1.27 (1.01 to 1.59)	4.15 (3.38 to 5.09)
Induced (n=2,542)	1.67 (1.47 to 1.91)	3.04 (2.23 to 4.15)	1.54 (1.26 to 1.87)	1.98 (1.46 to 2.67)	1.94 (1.52 to 2.49)	1.56 (1.21 to 2.01)	2.67 (1.98 to 3.60)
P for interaction	0.75	<0.001	0.27	0.70	0.26	0.24	0.02
34-36							
Spontaneous (n=36,446)	1.29 (1.24 to 1.35)	1.78 (1.55 to 2.04)	1.33 (1.24 to 1.42)	1.22 (1.09 to 1.36)	1.40 (1.29 to 1.52)	1.14 (1.05 to 1.25)	1.54 (1.36 to 1.74)
Induced (n=11,505)	1.27 (1.18 to 1.37)	1.95 (1.58 to 2.40)	1.17 (1.04 to 1.32)	1.19 (0.98 to 1.44)	1.42 (1.23 to 1.65)	1.18 (1.01 to 1.37)	1.50 (1.22 to 1.86)
P for interaction	0.71	0.48	0.08	0.83	0.83	0.73	0.85
37-38							
Spontaneous (n=164,319)	1.08 (1.05 to 1.11)	1.27 (1.16 to 1.38)	1.15 (1.11 to 1.20)	1.05 (0.98 to 1.11)	1.09 (1.04 to 1.14)	1.04 (0.99 to 1.09)	1.07 (1.00 to 1.16)
Induced (n=89,547)	1.01 (0.97 to 1.06)	1.09 (0.95 to 1.25)	0.97 (0.91 to 1.04)	0.99 (0.90 to 1.10)	1.06 (0.98 to 1.15)	1.00 (0.92 to 1.08)	1.04 (0.92 to 1.18)
P for interaction	0.008	0.06	<0.001	0.40	0.61	0.36	0.71
39-40							
Spontaneous (n=628,409)	Reference	Reference	Reference	Reference	Reference	Reference	Reference
Induced (n=80,663)	Reference	Reference	Reference	Reference	Reference	Reference	Reference
41							
Spontaneous (n=225,018)	0.99 (0.97 to 1.01)	0.97 (0.89 to 1.06)	0.98 (0.94 to 1.01)	0.97 (0.91 to 1.02)	1.02 (0.98 to 1.07)	0.97 (0.93 to 1.02)	1.01 (0.95 to 1.09)
Induced (n=27,307)	0.95 (0.90 to 1.01)	0.89 (0.73 to 1.09)	0.91 (0.83 to 1.00)	0.92 (0.79 to 1.07)	0.96 (0.85 to 1.08)	1.03 (0.92 to 1.15)	0.96 (0.80 to 1.15)
P for interaction	0.23	0.43	0.18	0.53	0.32	0.41	0.54

CI=confidence interval.

[a]Adjusted for maternal age at delivery, parity, country of birth, cohabiting status, body mass index during early pregnancy, smoking during pregnancy, diabetic and hypertensive diseases, calendar period of delivery, onset of labor, parental highest educational level, parental history of neurological or psychiatric disorder, infant's sex and birth weight for gestational age.

[b]At least one of motor, cognitive, epileptic, visual, or hearing impairment.

[c]Diagnosis of cerebral palsy, severe mental retardation, generalised epileptic disorder, or severe hearing or visual impairment.

Supplementary Table I. Association between gestational age (32 to 41 weeks) and neurodevelopmental outcomes among singleton live-born children without congenital malformations in Sweden 2001-2012 (N=1,057,792)

Gestational age (weeks)	Any impairment[a]	Any motor impairment	Any cognitive impairment	Any epileptic impairment	Any visual impairment	Any hearing impairment	Any severe or major impairment[b]
32-33 (n=6,125)							
Person-years	70,080	73,276	73,222	73,984	73,780	73,854	73,462
No. of cases (Rate[c])	668 (95.3)	167 (22.8)	271 (37.0)	116 (15.7)	155 (21.0)	156 (21.1)	154 (21.0)
HR (95% CI)[d]	1.73 (1.59 to 1.89)	4.83 (3.99 to 5.86)	1.80 (1.57 to 2.06)	1.99 (1.62 to 2.45)	1.62 (1.35 to 1.94)	1.35 (1.12 to 1.62)	3.51 (2.90 to 4.24)
34-36 (n=39,898)							
Person-years	466,914	484,410	480,702	484,171	482,120	482,353	484,617
No. of cases (Rate[c])	3,082 (66.0)	351 (7.3)	1,172 (24.4)	459 (9.5)	844 (17.5)	777 (16.1)	393 (8.1)
HR (95% CI)[d]	1.30 (1.25 to 1.36)	1.95 (1.72 to 2.20)	1.29 (1.21 to 1.38)	1.23 (1.10 to 1.36)	1.40 (1.30 to 1.52)	1.19 (1.10 to 1.29)	1.58 (1.40 to 1.77)
37-38 (n=213,699)							
Person-years	2,511,591	2,586,470	2,567,104	2,582,098	2,574,274	2,572,633	2,586,139
No. of cases (Rate[c])	13,176 (52.5)	1,092 (4.2)	5,081 (19.8)	1,941 (7.5)	3,412 (13.3)	3,479 (13.5)	1,314 (5.1)
HR (95% CI)[d]	1.08 (1.06 to 1.11)	1.24 (1.15 to 1.34)	1.13 (1.09 to 1.18)	1.05 (0.99 to 1.11)	1.09 (1.04 to 1.14)	1.04 (0.99 to 1.08)	1.07 (1.00 to 1.15)
39-40 (n=589,207)							
Person-years	6,899,814	7,083,502	7,038,145	7,067,398	7,051,166	7,044,484	7,079,034
No. of cases (Rate[c])	32,347 (46.9)	2,277 (3.2)	11,558 (16.4)	5,108 (7.2)	8,399 (11.9)	8,939 (12.7)	3,322 (4.7)
HR (95% CI)[d]	Reference	Reference	Reference	Reference	Reference	Reference	Reference
41 (n=208,863)							
Person-years	2,460,450	2,525,680	2,509,759	2,519,924	2,513,356	2,512,384	2,523,573
No. of cases (Rate[c])	11,413 (46.4)	807 (3.2)	4,029 (16.1)	1,797 (7.1)	3,037 (12.1)	3,099 (12.3)	1,233 (4.9)
HR (95% CI)[d]	0.98 (0.96 to 1.00)	0.94 (0.86 to 1.03)	0.96 (0.92 to 0.99)	0.98 (0.92 to 1.03)	1.02 (0.97 to 1.06)	0.96 (0.92 to 1.01)	1.03 (0.96 to 1.10)

HR=hazard ratio; CI=confidence interval.

[a]At least one of motor, cognitive, epileptic, visual, or hearing impairment.

[b]Diagnosis of cerebral palsy, severe mental retardation, generalised epileptic disorder, or severe hearing or visual impairment.

[c]Number with outcome per 10,000 person years.

[d]Adjusted for maternal age at delivery, parity, country of birth, cohabiting status, body mass index during early pregnancy, smoking during pregnancy, diabetic and hypertensive diseases, calendar period of delivery, parental highest educational level, parental history of neurological or psychiatric disorder, infant's sex and birth weight for gestational age.
